# Behavioral responses to smoking bans in local public places in China: A secondary dataset analysis of China Family Panel Studies, 2010–2020

**DOI:** 10.18332/tid/220283

**Published:** 2026-06-26

**Authors:** Wenjie Meng, Ying'er Ma, Yunqiang Fu, Ting Chen

**Affiliations:** 1 School of Public Health, Wuhan University of Science and Technology, Healthy Hubei Development and Social Progress Research Center of the Key Research Base of Humanities and Social Sciences, Wuhan, China; 2 School of Resources and Environmental Engineering, Wuhan University of Science and Technology, Wuhan, China

**Keywords:** difference-in-differences, smoking bans, smoking behavior

## Abstract

**INTRODUCTION:**

While public smoking bans help reduce smoking behavior, tobacco control in China is mainly managed locally. The effects of regional regulations on individual smoking are unclear. This study analyses public smoking bans across several cities to inform local and national tobacco control efforts.

**METHODS:**

This study is a secondary data analysis using the 2010–2020 China Family Panel Studies (CFPS) nationwide dataset. The study adopts an observational design covering a 10-year period from 2010 to 2020, with data collected biennially across waves. The study population comprised respondents, aged ≥16 years from the CFPS 2010–2020 waves, who resided in the 37 cities selected for this quasi-experimental design. Individuals were included if they had complete data on smoking behavior, demographic characteristics, and key socioeconomic variables.

**RESULTS:**

Local tobacco control regulations did not significantly affect smoking status. They were related to the amount smoked (β=0.374; 95% CI: 0.115–0.633; p=0.005), with a significant positive correlation and heterogeneity observed in the subgroup analysis. In Hangzhou, the smoking probability increased by 0.021 (95% CI: 0.013–0.030, p<0.001) after policy enactment, and daily cigarette consumption among smokers increased by 0.696 (95% CI: 0.507–0.886, p<0.001). In Tianjin, the smoking probability decreased by 0.040 (95% CI: -0.048 – -0.032, p<0.001), and daily cigarette consumption dropped by 1.532 (95% CI: -1.684 – -1.379, p<0.001). In Guangzhou, the policy had no effect on smoking probability but reduced daily cigarette consumption by 0.316 (95% CI: -0.507 – -0.124, p=0.002). The effects of tobacco control policies vary significantly across different cities.

**CONCLUSIONS:**

This study identifies substantial regional heterogeneity in the effects of local public-place smoking bans, supporting the need for national standardization and context-specific regulatory strategies.

## INTRODUCTION

Globally, smoking is the leading external cause of death^1^. In 2019, there were 1.14 billion current smokers worldwide, consuming tobacco products equivalent to 7.41 trillion cigarettes. The number of deaths caused by smoking reached 7.69 million, resulting in a loss of 200 million disability-adjusted life years. Among all smoking-related deaths, 86.9% were current smokers^2^. This public health crisis has prompted the international community to prioritize strengthening tobacco control as a key action goal^3^. As the world’s largest consumer of tobacco (with 308 million active smokers), China is facing an increasingly heavy burden of disease. Relevant studies have shown that the number of annual smoking-related deaths in China exceeded one million in the 2010s^4^ and is on a continuous upward trend. Forecasts indicate that without effective intervention measures, this value could rise to 2 million per year by the 2030s and reach 3 million per year by 2050, with related economic losses estimated at 0.9% of GDP^5^.

To this end, China has introduced several tobacco control strategies and policies. In November 2003, China became the 77th signatory to the World Health Organization Framework Convention on Tobacco Control (FCTC)^6^. In 2005, it ratified the WHO Framework Convention on Tobacco Control and gradually strengthened a series of national and local tobacco control policies^7,8^. At the local level, from 2009 to 2013, nearly half of China’s provincial capital cities passed smoking ban legislation^9^, including Shanghai, Hangzhou, Guangzhou, Tianjin, Lanzhou, and Beijing. These regional practices have accumulated important experience for national-level legislation^10-12^. However, most existing studies focus on the analysis of individual cities or policy texts^13^, and there is a lack of systematic comparisons of the enactment effects of policies in multiple cities, especially the quantitative analysis of the impact on individual smoking behaviors (smoking probability and smoking amount; smoking probability and intensity)^11,12^. Based on this, this study takes residents across various cities in China as the research subjects, with tobacco control policies in public places of each city as the core intervention variable. It uses the difference-in-differences model to systematically evaluate the impact of tobacco control legislation on residents’ smoking behavior in different cities, aiming to identify the policy effects at the micro level and provide a scientific basis and decision-making reference for optimizing the tobacco control policy system in China.

## METHODS

### Study design

This study is a secondary dataset analysis of the China Family Panel Studies (CFPS) 2010–2020, using a quasi-experimental framework with a staggered difference-in-differences (DID) design to evaluate the effects of local public smoking bans on individual smoking behaviors. The study covered the period from 2010 to 2020, corresponding to six waves of the CFPS survey (2010, 2012, 2014, 2016, 2018, and 2020), with a total observation period of 11 years.

To implement this design, six cities that promulgated local tobacco control regulations during the study period were selected as the treatment group: Shanghai, Hangzhou, Guangzhou, Tianjin, Lanzhou, and Beijing. These cities enacted their bans in 2010, 2010, 2010, 2012, 2014, and 2015, respectively, and regularly released enforcement reports, ensuring policy intensity and compliance monitoring (Supplementary file Table 1). To construct a valid counterfactual, 31 prefecture-level cities without such legislation were selected as the control group using propensity score matching based on socioeconomic characteristics. By comparing the intervention and control groups before and after the policy was enacted, the policy’s net effect was identified.

### Study population

The study population comprised respondents aged ≥16 years from the CFPS 2010–2020 waves who resided in the 37 cities selected for this quasi-experimental design. Individuals were included if they had complete data on smoking behavior, demographic characteristics, and key socioeconomic variables. Observations with missing values for the key variables were excluded. After applying these criteria, the final analytical sample consisted of 20301 individuals, contributing 70909 person-year observations across the six survey waves.

### Data source

The data are from the CFPS, collected by the Center for Chinese Social Sciences Survey (ISSS) at Peking University^14^. The CFPS tracks and collects data at the individual, family, and community levels to reflect changes in China’s society, economy, population, education, and health, and provides a data basis for academic research and public policy analysis. The baseline survey was officially launched in 2010 and followed up every two years thereafter. This study selected data from six periods: 2010, 2012, 2014, 2016, 2018, and 2020. This database covers information at three levels: individuals, families, and communities, including variables such as income level, work status, education level, cognitive ability, non-cognitive ability, individual characteristics, and social characteristics.

### Ethics

This study involved secondary analysis of de-identified publicly available data from the China Family Panel Studies (CFPS), which were collected by the Center for Chinese Social Sciences Survey (ISSS) at Peking University. The original data collection of CFPS has been approved by the Research Ethics Committee of Peking University (IRB0000105213074). The participants provided written informed consent to participate in this study. Moreover, informed consent was obtained from literate participants and legal guardian(s)/next of kin of illiterate participants. All research methods were performed in accordance with relevant guidelines and regulations, and complied with the requirements of the Declaration of Helsinki.

### Variables

*Outcome variable*

The primary outcome variables were individual smoking status and daily smoking amount. Smoking status was measured using the standard question from the China Family Panel Studies (CFPS): ‘Have you smoked at least one cigarette in the past 30 days?’ with dichotomous yes/no responses; the binary variable was coded as 1 for respondents who smoked at least one cigarette in the past 30 days and 0 otherwise, reflecting recent smoking behavior rather than regular or lifetime smoking. Daily smoking amount was defined as the usual daily number of cigarettes consumed by current smokers in the past 30 days, consistent with the 30-day reference period used to define smoking status in the CFPS questionnaire.

*Explanatory variable*

The explanatory variable is a binary indicator of whether a city had enacted a formal smoking control regulation in public places. Enactment dates were identified from official regulatory documents published on municipal government websites. Specifically, six cities adopted such policies during 2010–2020: Shanghai, Hangzhou, and Guangzhou in 2010; Tianjin in 2012; Lanzhou in 2014; and Beijing in 2015. The policy exposure variable was coded as 1 for observations in examined cities in and after the year of enactment, and 0 otherwise.

### Covariates

The covariates included demographic characteristics: age, gender, residential area, marital status (married/cohabiting, not married/divorced/separated), education level (primary school or lower, middle school/high school/vocational high school, junior college or higher), ln (average household income), chronic disease, household smoking, and alcohol use.

### Statistical analysis

*Descriptive statistics*

Descriptive statistics were used to present the distribution of all variables, including frequencies and percentages for categorical variables, and means and standard deviations for continuous variables. Before conducting descriptive analyses, missing data were imputed, where possible. Specifically, for current smokers with missing daily consumption, the value was replaced by the wave-specific mean of non-missing amounts. Age was linearly interpolated within individuals across survey waves using the *ipolate* command; observations with age still missing after interpolation were dropped. Education level was forward-filled using the individual’s own value from the most recent prior wave (lag-2 and lag-4). Urban residence was similarly carried forward from the previous wave when possible; otherwise, the observation was excluded. Household income was constructed by summing multiple income sources (farm, business, property, welfare, and other incomes); if the reported total was missing, it was replaced by this sum. Any remaining zero or missing values were imputed with the sample mean of positive household incomes. Per capita equivalent income was then calculated as household income divided by family size raised to the power of 0.56 and log-transformed. Family smoking status, derived from individual smoking reports, was set to zero if missing. Observations with missing marital status were excluded because no reliable imputation was feasible. To mitigate the influence of outliers, continuous variables – daily smoking amount, age, and per capita household income were winsorized at the 1st and 99th percentiles using the *winsor2* command in Stata.

*Baseline regression*

On the basis of the binary characteristics (whether smoking or not) and continuous characteristics (average daily smoking amount) of smoking behavior, a multi-time point difference-in-differences (staggered DID) model was constructed, and the effect of smoking control regulations in public places was estimated via the two-way fixed effects (TWFE) framework. For the binary dependent variable (smoking status), the choice of a linear probability model over the logit/probit nonlinear model is mainly based on the following methodological considerations. First, comparing the parameter estimates in nonlinear models with different covariate sets can be very complex and challenging. Second, integrating fixed effects into logit and probit models may lead to methodological issues^15^.

*Robustness check*

Although the TWFE model is a common method for estimating multiperiod DID, estimation bias may arise when there is temporal heterogeneity in the treatment effect^16^. Additionally, predicted values from the linear probability model may exceed the range 0–1, leading to biased results.

To address the above issues, robustness tests were conducted using the CSDID method by Callaway and Sant’Anna^17^. This method matches the intervention group with a control group unaffected by the policy. It estimates policy effects across periods, aggregates them with weights, and controls for temporal heterogeneity and model selection bias. This approach enhances the reliability of the conclusion.

*Software and statistical threshold*

All statistical analyses were performed using Stata 17.0 (StataCorp LLC, College Station, TX, USA). A two-sided p<0.05 was considered statistically significant.

## RESULTS

### Descriptive statistics

Table 1 presents the core characteristics and smoking behavior distributions of the population in 37 sample cities (including 6 intervention cities and 31 control cities) from 2010–2020. Overall, the smoking rate and amount of smoking among the population in 37 cities from 2010–2020 showed a downward trend. The individual smoking rate decreased from 28.44% in 2010 to 25.24% in 2020, and average daily cigarette consumption decreased from 4.44 (SD=8.69) to 3.34 (SD=7.00) cigarettes per day.

**Table 1:** Descriptive statistics of demographic characteristics and smoking behaviors among participants in 37 sample cities (6 treatment + 31 control) from the China Family Panel Studies (CFPS), 2010–2020

Variables	2010 (N=14438) n (%)	2012 (N=12912) n (%)	2014 (N=12520) n (%)	2016 (N=11783) n (%)	2018 (N=11191) n (%)	2020 (N=8065) n (%)
**Smoking status**						
Non-smoker	10333 (71.56)	9284 (71.90)	9203 (73.51)	8702 (73.85)	8148 (72.81)	6029 (74.76)
Smoker	4105 (28.44)	3628 (28.10)	3317 (26.49)	3081 (26.15)	3043 (27.19)	2036 (25.24)
Cigarettes per day, mean ± SD	4.44 ± 8.69	4.38 ± 8.64	4.08 ± 8.30	3.92 ± 8.08	3.95 ± 8.01	3.34 ± 7.00
**Age** (years), mean ± SD	46.21 ± 16.78	46.72 ± 16.92	47.37 ± 16.98	47.50 ± 17.21	48.56 ± 17.04	47.24 ± 16.90
**Gender**						
Female	7442 (51.54)	6651 (51.51)	6469 (51.67)	5999 (50.91)	5702 (50.95)	4066 (50.42)
Male	6996 (48.46)	6261 (48.49)	6051 (48.33)	5784 (49.09)	5489 (49.05)	3999 (49.58)
**Residential area**						
Urban	9282 (64.28)	7965 (61.69)	7852 (62.72)	7495 (63.61)	7314 (65.36)	5189 (64.33)
Rural	5156 (35.72)	4947 (38.31)	4668 (37.28)	4288 (36.39)	3877 (34.64)	2876 (35.67)
**Marital status**						
Married/cohabiting	11438 (79.22)	10337 (80.06)	9993 (79.82)	9322 (79.11)	8940 (79.89)	6281 (77.88)
Not married/divorced/separated	3000 (20.78)	2575 (19.94)	2527 (20.18)	2461 (20.89)	2251 (20.11)	1784 (22.12)
**Education level**						
Primary school or lower	5882 (40.74)	5577 (43.19)	5263 (42.04)	4675 (39.68)	4014 (35.87)	2328 (28.86)
Middle school/high school/vocational high school	7012 (48.56)	5912(45.79)	5794 (46.28)	5438 (46.15)	5428 (48.50)	4117 (51.05)
Junior college or higher	1544 (10.70)	1423(11.02)	1463 (11.68)	1670 (14.17)	1749 (15.63)	1620 (20.09)
**Ln (Average household income),** mean ± SD	9.61 ± 1.02	9.67 ± 1.27	9.97 ± 1.14	10.11 ± 1.21	10.43 ± 1.22	10.54 ± 1.12
**Chronic disease**						
Absence	13207 (91.47)	11606 (89.89)	11037 (88.15)	10415 (88.39)	9756 (87.18)	7113 (88.20)
Presence	1231 (8.53)	1306 (10.11)	1483 (11.85)	1368 (11.61)	1435 (12.82)	952 (11.80)
**Household smoking**						
Household with smoker(s)	8334 (57.72)	7296 (56.51)	7379 (58.94)	6943 (58.92)	6802 (60.78)	5348 (66.31)
Smoke-free household	6104 (42.28)	5616 (43.49)	5141 (41.06)	4840 (41.08)	4389 (39.22)	2717 (33.69)
**Alcohol use**						
Non-drinker	12075 (83.63)	10739 (83.17)	10451 (83.47)	9927 (84.25)	9325 (83.33)	6914 (85.73)
Drinker	2363 (16.37)	2173 (16.83)	2069 (16.53)	1856 (15.75)	1866 (16.67)	1151 (14.27)

Data source: 2010–2020 China Family Panel Studies (CFPS). Data processing: missing values were interpolated; 1% Winsorized tail reduction was applied to continuous variables (cigarettes per day, ln (average household income)) to eliminate extreme value interference.

### Baseline regression and parallel trends

*Baseline regression*

Table 2 reports the benchmark regression results from the two-way fixed effects (TWFE) model. Smoking status indicates the effect of tobacco control regulations on smoking status, and cigarettes per day indicates the effect on average daily smoking consumption. The results revealed that, regardless of whether control variables were included, there was no significant association between tobacco control policy and smoking status (p>0.05). After adjusting for covariates, the coefficient for smoking status was 0.011 (95% CI: -0.002–0.024, p=0.086), indicating that the policy did not significantly change the population’s smoking probability. However, public-place tobacco control policy was significantly positively correlated with average daily smoking consumption (β=0.374; 95% CI: 0.115–0.633, p=0.005), indicating that average daily smoking consumption increased by 0.374 cigarettes after policy enactment.

**Table 2:** Estimated effects, robustness check, and gender-specific heterogeneous effects of municipal public smoking bans on smoking status and daily cigarette consumption among residents in 37 sample cities (6 treatment + 31 control) from the China Family Panel Studies (CFPS), 2010–2020

*Analysis*	*Variable*	*β*	*95% CI*	*p*
**Baseline regression**	Smoking status	0.010^a^	-0.007–0.026	0.244
		0.011^b^	-0.002–0.024	0.086
	Cigarettes per day	0.375^a^	0.067–0.682	0.017
		0.374^b^	0.115–0.633	0.005
**Robustness check**	Smoking status	0.010^c^	0.000–0.020	0.048
	Cigarettes per day	0.382^c^	0.148–0.617	0.001
**Subgroup analysis by gender**	Smoking status	0.019^d^	-0.006–0.044	0.134
		0.005^e^	-0.002–0.012	0.165
	Cigarettes per day	0.716^d^	0.208–1.224	0.006
		0.080^e^	0.009–0.151	0.028

The table presents the estimated effects of public smoking bans on smoking-related outcomes. Baseline regression was conducted using the two-way fixed effects (TWFE) staggered difference-in-differences (DID) model. The robustness check verifies the reliability of TWFE results with the 2 years pre-policy as the reference group. Subgroup analysis by gender shows gender-stratified estimates based on the TWFE model.

a Without control variables.

b With control variables.

c Estimated by the CSDID method.

d Male subgroup.

e Female subgroup.

All results reported as p<0.001 correspond to an actual output of 0.000 in statistical software, in accordance with standard academic reporting conventions for extremely small p-values.

*Parallel trends test*

**Figure 1:**
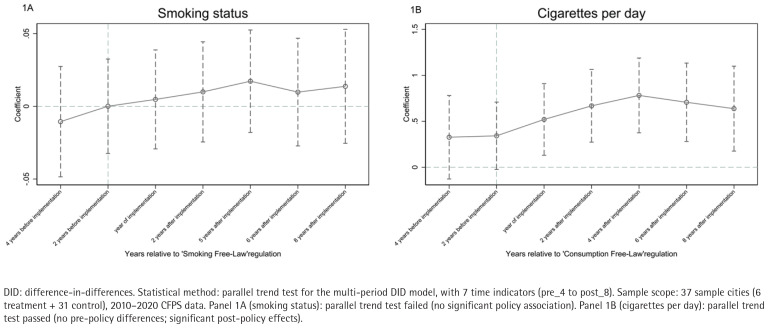
Parallel trend test for the effects of public smoking bans on residents’ smoking behaviors (two-way fixed effects model), China Family Panel Studies (CFPS), 2010–2020

The prerequisite for using the difference-in-differences model to evaluate the impact of local public smoking control policies on smoking intensity is that, before the policy’s enactment, the smoking behavior-related indicators of the treatment and control groups should exhibit a common time trend. On this basis, it is necessary to use the parallel trend test to examine the multi-time point difference-in-differences model in this study. According to the time when different cities enacted the smoking control policy in public places, the sample intervals were divided into the first 4 years, the first 2 years, the year of enactment, the last 2 years, the last 4 years, the last 6 years and the last 8 years of enactment of the smoking control policy in public places, thereby constructing 7 indicator variables. The results of the parallel trend test for 4 years before enactment, 2 years before enactment, the year of enactment, 2 years after enactment, 4 years after enactment, 6 years after enactment, and 8 years after enactment are shown in Figure 1.

Before and after policy enactment, no significant differences in individual smoking status were observed between pilot and non-pilot cities in the sample. The parallel trend assumption was not satisfied, and tobacco control regulations were not significantly associated with individual smoking status (all p>0.05). It was not significant in the four years before the policy enactment, the two years before the policy enactment, or the current period of policy enactment, indicating that before the enactment of the smoking control policy in public places, there was no significant difference in the average daily smoking amount between individuals in the pilot cities and nonpilot cities in the sample (all p>0.05). This result indicates that in the absence of policy intervention, the development trend of the average daily smoking number of individuals in the experimental group and the control group remained consistent. Two years after the policy enactment, four years after the policy enactment, six years after the policy enactment, and eight years after the policy enactment, positive and significant differences began to appear between the experimental group and the control group (all p<0.05), the policy enactment effect began to emerge, and the parallel trend test was passed.

### Robustness check

China’s tobacco control actions are managed on a territorial basis, with each city independently determining its local tobacco control policies and adjustment times. The adjustment of smoking control policies in public places at different times in different regions conforms to the typical characteristics of the multiperiod difference-in-differences method. When the treatment effect changes over time, the estimation results of the traditional bidirectional fixed-effect model will be biased by the influence of the ‘bad control group’. Therefore, this study uses CSDID to test the impact of smoking control regulations in public places on individual smoking intensity, and the regression results are as follows.

**Figure 2:**
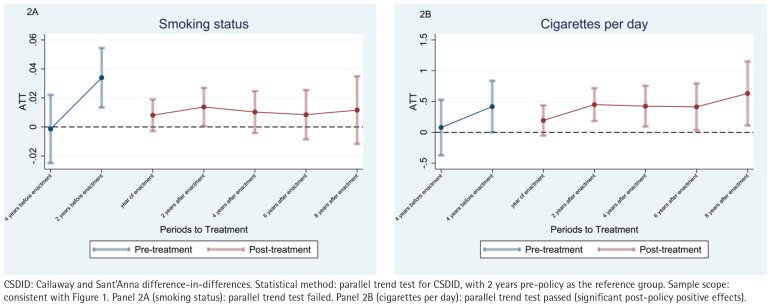
Parallel trend test for public smoking ban effects on smoking behavior using the CSDID method, China Family Panel Studies (CFPS), 2010–2020

Table 2 shows that smoking control regulations in public places are significantly positively correlated with smoking status (β=0.010; 95% CI: 0.000–0.020, p=0.048) and average daily smoking consumption (β=0.382; 95% CI: 0.148–0.617, p=0.001). However, the parallel trend test shown in Figure 2 indicates that the smoking status outcome failed to satisfy the parallel trend assumption, while daily cigarette consumption satisfied the assumption. These results are consistent with the baseline regression findings presented earlier. Taking the two years before treatment as the benchmark group, there was no significant correlation between the policies of the four years before treatment, the two years before treatment, and the year of treatment, and the average daily smoking consumption (all p>0.05). However, in the second, fourth, sixth, and eighth years after treatment, the policy effect was significantly positive at the 5% level (all p<0.05), indicating that the research design meets the parallel trend test and that the tobacco control policy is significantly positively correlated with average daily smoking consumption.

### Heterogeneity analysis

*Grouping by gender*

To investigate the impact of smoking control regulations in public places across different gender groups, this study conducted a gender-heterogeneous analysis, and the results are shown in Table 2. Smoking control regulations in public places have no significant effect on whether men or women smoke (p>0.05), but are significantly positively correlated with the average daily smoking consumption. After the enactment of local tobacco control regulations, the average daily smoking consumption among men increased by 0.716 cigarettes (95% CI: 0.208–1.224, p=0.006), with an increase of 0.080 cigarettes in females (95% CI: 0.009– 0.151, p=0.028). This result is consistent with the overall trend, and the increase in smoking among men is greater.

*Grouping by city*

Tobacco control regulations in public places have no significant connection with whether an individual smokes, but are significantly positively correlated with the average daily smoking consumption of an individual. Given that the enactment intensity of tobacco control policies varies across cities, these policies have different impacts on individual smoking behaviors. Among the six cities included in the analysis, the tobacco control policies of Hangzhou, Lanzhou, and Shanghai are significantly positively correlated with the number of individual smokers and the average daily smoking consumption. The tobacco control policies of Beijing and Tianjin are negatively correlated with smoking status. The tobacco control policy in Guangzhou has no significant correlation with whether an individual smokes. The tobacco control policies in Guangzhou, Beijing, and Tianjin are significantly negatively correlated with the average daily smoking consumption. There is significant heterogeneity in the impact of tobacco control policies in public places in different cities on whether an individual smokes and the average daily smoking consumption.

After the enactment of the tobacco control policy in Hangzhou, the average probability of individuals becoming smokers increased by 0.021 (95% CI: 0.013–0.030, p<0.001), and the average daily smoking consumption of smokers significantly increased by 0.696 cigarettes (95% CI: 0.507–0.886, p<0.001). The tobacco control policy in Guangzhou has no significant correlation with smoking status (β= -0.007; 95% CI: -0.015–0.002, p=0.119), but it significantly reduces the average number of cigarettes smoked daily by 0.316 cigarettes (95% CI: -0.507 – -0.124, p=0.002). After the enactment of the tobacco control policy in Beijing, the average probability of individuals becoming smokers decreased by 0.011 (95% CI: -0.018 – -0.004, p=0.004), and the average daily smoking consumption of smokers significantly decreased by 0.213 cigarettes (95% CI: -0.370 – -0.057, p=0.009). After Lanzhou enacted the tobacco control policy, the average probability of individuals becoming smokers increased by 0.015 (95% CI: 0.007–0.024, p=0.001), and the average daily smoking consumption of smokers significantly increased by 0.522 cigarettes (95% CI: 0.355–0.689, p<0.001). After the enactment of the tobacco control policy in Tianjin, the average probability of individuals becoming smokers decreased by 0.040 (95% CI: -0.048 – -0.032, p<0.001), and the average daily smoking consumption of smokers significantly decreased by 1.532 cigarettes (95% CI: -1.684 – -1.379, p<0.001). After the enactment of the tobacco control policy in Shanghai, the average probability of individuals becoming smokers increased by 0.022 (95% CI: 0.014–0.030, p<0.001), and the average daily smoking consumption of smokers significantly increased by 0.685 cigarettes (95% CI: 0.497–0.872, p<0.001).

## DISCUSSION

In this study, the difference-in-differences method was used to explore the impact of smoking control regulations on individual smoking behavior in six cities in China that implemented public-place smoking control policies from 2010 to 2020, revealing the complexity and heterogeneity of the influence of local smoking control policies on smoking behavior.

Research shows that, on the whole, local public place tobacco control regulations have not reduced the smoking rate among the population; instead, they have increased the average daily smoking consumption, which is contrary to the theoretical expectation that ‘tobacco control policies reduce smoking behavior’. The finding that local public smoking control regulations have not reduced the smoking rate among the population is consistent with evidence from the United States, Australia, and other European countries^18-20^. The increase in the average daily smoking consumption is consistent with the findings of Luo and Zhao^21^ and Wen et al.^22^. This might be because public regulations significantly limit smoking opportunities and time for smokers in specific places, such as restaurants, bars, and offices. To maintain their original nicotine intake levels or meet their habitual needs, some smokers tend to do so in unrestricted private spaces (such as at home or in the car) or permitted outdoor areas^23^. Compensation is made by increasing the amount of smoking per session (such as smoking deeper or longer) or by increasing the frequency of smoking, which leads to an increase in the total daily smoking consumption. The study of Adda and Cornaglia^24^ found that tobacco control interventions lead smokers to reduce the number of cigarettes consumed but increase smoking intensity per cigarette to maintain nicotine intake, directly confirming the existence of such compensatory smoking behavior. This ‘loss-compensation’ mentality is a common pattern in behavioral economics^25^.

In addition, subgroup analyses of the effects of tobacco control regulations by gender and city were conducted. The results revealed that the enactment of local tobacco control regulations had no significant effect on whether men or women smoked, consistent with the findings of Yang et al.^26^ who similarly found that local tobacco control regulations did not significantly influence smoking initiation or cessation rates for either men or women. But significantly increased the average daily smoking consumption of both groups, and the increase in smoking consumption among men was greater. This might be directly related to the higher smoking rate among men and the more direct impact of restrictions in public places^10,27^.

There are differences in the policy enactment effects across the six cities, consistent with the findings of Fong et al.^28^, and these differences may be due to varying policy enactment intensities and different supporting measures. Beijing and Tianjin have adopted a dual-track model of supervision and support, deeply integrating the MPOWER measures of ‘Protect’ and ‘Offer’^29^. Through intensive law enforcement (such as frequent inspections and heavy fines), they have reduced smoking in public places. At the same time, they have provided support measures such as community smoking cessation clinics and hotline consultations to help smokers reduce their cigarette consumption or even quit smoking. Ultimately, this has led to a decrease in both the smoking rate and the cigarette consumption. Especially in Tianjin, the average daily cigarette consumption still decreased by 1.53 cigarettes eight years after the policy was implemented, indicating that long-term, systematic intervention can stabilize the policy’s effect. The policies in Hangzhou, Shanghai, and Lanzhou are focused on high-intensity regulation, but they lack support for quitting smoking and alternative places (such as designated smoking areas), which leads smokers to turn to private places and increase their cigarette consumption^30^. For example, Shanghai, as an economically developed city, has strict regulations on public places, but lacks restrictions on smoking at home. As a result, smokers are more likely to engage in compensatory smoking in a family environment. The mixed policy effects in Guangzhou reflect the uneven regulatory coverage. In some areas (such as hospitals and schools), strict law enforcement has reduced the amount of smoking^31^. However, the lax supervision of catering and entertainment venues failed to significantly reduce the smoking rate, ultimately resulting in a decrease in the amount of smoking but an unchanged smoking rate. The successful experiences of Beijing and Tianjin confirm the importance of Social Cognitive Theory, which posits that behavioral change results from interactions among personal, environmental, and behavioral factors^32^. By combining enforcement and cessation services, Beijing and Tianjin implemented multidimensional interventions to address smoking behavior, effectively breaking the ‘restriction-compensation’ cycle. A systematic review and meta-analysis of population-level tobacco control interventions also indicates that comprehensive measures are significantly more effective than single regulatory measures^33^.

### Limitations

Although this research provides valuable evidence on the impact of local public smoking bans on individual smoking behavior, several limitations remain. First, potential unmeasured confounding and omitted variable bias cannot be fully eliminated. Specifically, concurrent unmeasured tobacco control activities (e.g. anti-smoking media campaigns, public education initiatives) may coincide with the enactment of local smoking bans and independently influence smoking behavior. Second, the analysis relies on information about the enactment of local anti-smoking regulations rather than direct measures of actual policy enactment or enforcement intensity, which makes it difficult to determine whether the policies translated into substantive on-the-ground interventions. In addition, enforcement intensity and regulatory scope likely varied across cities, which limits the ability to distinguish whether observed differences reflect differential policy effectiveness or heterogeneity in enactment strength. Third, the findings may have limited external validity because the analysis focuses on large urban cities and may not be generalizable to smaller cities or rural settings. Fourth, potential spillover effects across cities, such as the displacement of smoking behavior or mobility of smokers to areas without corresponding interventions, cannot be ruled out. Finally, reliance on self-reported smoking data may introduce misclassification bias.

## CONCLUSIONS

In China, there are significant differences in the enactment effects of tobacco control policies among different cities. These findings highlight the limitations of local public place control, and it is urgent to formulate national laws and regulations on tobacco control in public places to increase the consistency and effectiveness of tobacco control policies^34^. Moreover, precise governance plans should be designed based on current conditions across different cities, and the enactment of smoking control policies in public places should be strengthened^35^. Only in this way can long-term and effective governance of smoking behavior be achieved.

## Supplementary Material



## Data Availability

The data supporting this research are available from the following sources: The China Family Panel Studies (CFPS) database, hosted by the Center for Chinese Social Sciences Survey (ISSS) at Peking University. The data can be accessed via the official CFPS website: http://www.isss.pku.edu.cn//. This study utilized data from six waves of the CFPS (2010, 2012, 2014, 2016, 2018, and 2020).
