# Supplementary material for: Behavioral responses to smoking bans in local public places in China: A secondary dataset analysis of China Family Panel Studies, 2010–2020
Source: Tob Induc Dis. 2026 Jun 26;24:10.18332/tid/220283. doi: 10.18332/tid/220283 (PMC13312264; doi:10.18332/tid/220283)
Supplement: Supplementary file 1 [file TID-24-96-s1.pdf]

Supplementary Table 1 Timing of municipal public smoking regulation enactment in six Chinese cities included in the China Family Panel Studies (CFPS), 2010–2020

City	Regulation	Effective Date
Shanghai	Regulation of Shanghai Municipality on Smoking Control in Public Places	2010.03
Hangzhou	Regulation of Hangzhou Municipality on Smoking Control in Public Places	2010.03
Guangzhou	Regulation of Guangzhou Municipality on Smoking Control	2010.09
Tianjin	Regulation of Tianjin Municipality on Tobacco Control	2012.05
Lanzhou	Regulation of Lanzhou Municipality on Smoking Control in Public Places	2014.01
Beijing	Beijing Regulation on Smoke-Free Public Places	2015.06

Data source: Regulatory texts from official municipal government websites.

This content has been provided by the author(s) and has not been reviewed, verified, or endorsed by European Publishing. It may not have undergone peer review. The views, opinions, and recommendations expressed are solely those of the author(s) and do not necessarily reflect the position of European Publishing. European Publishing accepts no responsibility or liability for any consequences arising from the use of, or reliance on, this content.

© 2026 Meng W. et al. DOI:10.18332/tid/220283
